# Effect of respiratory microflorae colonization on short and long‐time outcomes of respiratory syncytial virus infection in children: A scoping review

**DOI:** 10.1002/pdi3.97

**Published:** 2024-07-16

**Authors:** Lidan Gan, Enmei Liu, Yu Deng

**Affiliations:** ^1^ Department of Respiratory Medicine Children's Hospital of Chongqing Medical University Chongqing China; ^2^ Ministry of Education Key Laboratory of Child Development and Disorders Children's Medical Big Data Intelligent Application Center Department of Respiratory Medicine Chongqing China; ^3^ National Clinical Research Center for Child Health and Disorders Chongqing China

**Keywords:** childhood asthma, disease severity, microflorae, recurrent wheezing, respiratory syncytial virus

## Abstract

Respiratory syncytial virus (RSV) is an essential cause of lower respiratory tract infection in children under 2 years of age, especially under 6 months. In decades, studies have shown that the respiratory tract microflorae with RSV infection were related to disease severity and played a role in the development of recurrent wheezing, but the effect of respiratory microflorae on RSV infection are still underestimated. This study aims to conclude the effect of respiratory microflorae colonization on RSV infectious disease severity and recurrent wheezing and provide suggestions for future research directions from the perspective of respiratory tract florae. We conducted a scoping review. Studies were eligible if they reported on the effect of microflorae on RSV infectious diseases among children. We exacted the following information: title, publication time, first author's country, and article type. We finally included 33 articles in this scoping review. The number of studies rapidly increased since 2013 and the highest number of hospitalizations were reported in children <2 years. More than half (69.70%) were conducted in America and most studies are original studies (57.58%). The Review highlighted that the respiratory microflorae played an important role in RSV infectious disease severity and recurrent wheezing. We found that *Streptococcus pneumoniae* (S.pn), *Haemophilus influenza* (HI), *Moraxella catarrhalis* (M.ca), and *Staphylococcus aureus* (SA) were the dominant profiles in children with RSV infection. Understanding the respective role of respiratory microflorae on RSV infection and its mechanisms would improve prevention and treatment strategies from the perspective of microflorae.

## 1 INTRODUCTION

Respiratory syncytial virus (RSV) is the most common viral pathogen, and most children experience RSV infection within 2 years of age.^1^, ^2^ Clinical manifestations may vary from mild to severe format which requires admission to hospital and eventually pediatric intensive care unit admission.^3^ About 3% of children with RSV infection require hospitalization, and early RSV infection is a risk factor for the later development of recurrent wheezing or asthma.^4^ Severe RSV infectious diseases may require prolonged hospitalization with a negative economic influence on society. Preterm, congenital heart disease, bronchopulmonary, malnutrition,^5^ immunodeficiency, and neuromuscular disease are known risk factors for RSV infection at present.^6^ Respiratory virus infection can only be treated symptomatically, and the Food and Drug Administration (FDA)‐approved vaccine against RSV infection has not yet been marketed.^7^

The respiratory tract is not sterile, and the bacterial community composition of the respiratory tract differs across anatomical niches.^8^ Data shows that microflorae in different anatomical niches have different physiological environments (e.g., pH, O_2_ concentration, size of gas particles), which results in variations in microbial community structure along the respiratory tract in terms of abundance and diversity. Early interactions between respiratory viruses and respiratory florae might modulate host immune responses and subsequently contribute to disease severity and later development of recurrent wheezing or asthma in children.^9^ Streptococcus pneumoniae, Haemophilus influenzae, Moraxella catarrhalis and Staphylococcus aureus (S.pn, HI, M.ca and SA) are the dominant microflorae in the respiratory tract. Many studies have investigated that S.pn, HI and M.ca are the risk factors for RSV disease severity and childhood asthma or recurrent wheezing,^10^, ^11^, ^12^, ^13^, ^14^, ^15^ but other studies got the opposite conclusion.^3^, ^16^ The “window of opportunity” of M.ca suggests that colonization after 3 months was beneficial for respiratory tract infection (RTI). In contrast, colonization before 1 month was non‐beneficial for RTI.^4^ It is unknown if specific bacterial colonization contributes to RSV infectious disease severity and the development of asthma or recurrent wheezing in RSV‐infected children. The scoping review aims to systematically review the existing literature and identify the knowledge gaps in the context of the relationship between airway flora and outcomes of RSV infection in children.

Scoping reviews are a useful tool in the increasing arsenal of evidence synthesis approaches. It followed recommendations set out in the Preferred Reporting Items for Systematic Reviews and Meta‐Analyses extension for Scoping Reviews (PRISMA‐SCR). The protocol was prospectively registered with the Open Science Framework (https://osf.io/s7jz2) and the registration DOI is 10.17605/OSF.IO/S7JZ2.

## 2 METHOD

### 2.1 Search strategy

We systematically searched databases including PubMed, EMBASE, Web of Science, Cochrane Library, China Biology Medicine (CBM), China National Knowledge Infrastructure (CNKI) and WanFang Data with the Mesh terms “Respiratory Syncytial Virus*” OR “Chimpanzee Coryza Agent*” OR “RSV” and “Microbiota*” OR “Microbial Community*” OR “Microbial Community Composition*” OR “Microbial community Structure*” OR “Microbiome*” OR “Microorganism*” OR “Microbiology” OR “Germ” OR “Microbial” OR “microbe*” OR “bacteria” OR “flora” OR “microflora”, unlimited time and language. Besides, we also supplementarily searched the official website of WHO (https://www.who.int), Google Scholar (https://scholar.google.hk), and two preprint servers, including BioRxiv (https://www.biorxiv.org/) and medRxiv (https://www.medrxiv.org/).

### 2.2 Inclusion and exclusion criteria

#### 2.2.1 Criteria for bacterial infection and colonization

Whether the bacteria detected in respiratory tract are pathogenic should be closely combined with clinical data. If the patient has elevated body temperature, cough and sputum, increased blood white blood cell count, neutrophil count, C‐reactive protein and procalcitonin, and new pulmonary infiltrating image in imaging examination and other manifestations of lung infection, it is suggested that the pathogen is pathogenic. If the clinical symptoms and laboratory indicators of infection were improved after anti‐infective treatment, and the number of target bacteria at the infection site was reduced, it was the pathogenic bacteria. On the contrary, it may be polluting bacteria or colonizing bacteria. If it is judged to be pathogenic, it should be given the corresponding anti‐infective treatment, if it is judged to be colonizing bacteria, anti‐infective treatment is not required.^17^, ^18^, ^19^

#### 2.2.2 Inclusion criteria

All retrieved papers that reported respiratory microflorae and RSV infection in children (≤18‐years‐old) were included.

#### 2.2.3 Exclusion criteria

Duplicates.
Studies that were unrelated to the field of respiratory microflorae and RSV infection.
The study population was adults, animals and cells.
Bacterial infection was clear.
Full text not available (such as studies inaccessible for downloading and conference abstracts without data).
Articles failed to access the full text after contacting the authors within 14 days.

### 2.3 Article selection and data extraction

Two researchers independently screened literature using the EndNote citation management software and disagreements were resolved by consensus after consultation. We recorded information using a standardized extraction table. Researchers used the inclusions and exclusions criteria first to screen the studies' titles and abstracts and excluded irrelevant literature. Then, the full text of the literature was reviewed to include the final eligible studies. Finally, the reasons for exclusion were recorded. We extracted the following information: title, publication date, first author's country, and type of article for every study. For original articles and abstracts with data, title, publication time, first author's country, study type, way of bacteria detection, age of included children, and outcome were extracted. The details are shown in the Supplementary Table.

### 2.4 Data analysis

We conducted a descriptive analysis of the included literature. The pictures were scanned by Microsoft Excel 2016.

## 3 RESULTS

### 3.1 Search results

**FIGURE 1:**
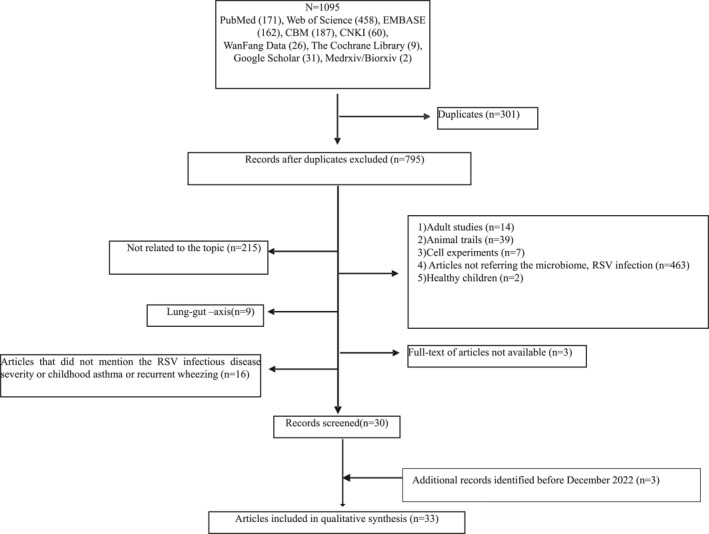
Flowchart of the selection process for the scoping review of respiratory tract microbiota with respiratory syncytial virus infection articles/studies and results. CBM, China Biology Medicine; CNKI, China National Knowledge Infrastructure.

A total of 1096 papers were retrieved, 301 of which were excluded as duplicates. Title and abstract screening were conducted for the remaining 795 papers, 215 of which were excluded because of being unrelated to the topic. Fourteen adult studies, 39 animal trials, 7 cell experiments, 463 studies without referring the microflora and RSV infection, 2 studies about the composition of the microflora in healthy children and the articles that did not mention the effect of microflora on RSV infectious disease severity, childhood asthma or recurrent wheezing were excluded. After contacting the authors, we failed to access the full text for three pieces. Finally, 33 papers were included. The flow chart of the selection process is shown in Figure 1.

### 3.2 Characteristics of included studies

**FIGURE 2:**
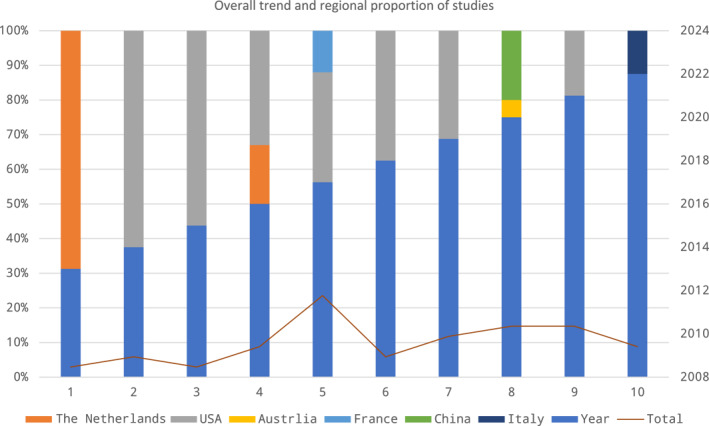
Overall trend and regional distribution in the included studies.

Of the 33 articles, there are five types of studies (prospective study, abstract with data, review, letter and editorial), and more than half are original articles (prospective studies) (57.58%). The highest number of hospitalizations included were <2 years of age, especially <1 year of age. The total amount of published studies related to the topic in children showed a rapid increase over the past decade. More studies originated from the USA (69.70%) and other developed countries (Figure 2). More information about the withdrawable data was scanned in the Supplementary Table.

### 3.3 Dominated microflora profiles with RSV infection in the included studies

Due to integrity of the research data, we selected 19 original studies out of 33 articles. Almost all sample sources were from nasopharyngeal aspirates (NPA). After RSV invaded the respiratory tract, bacterial diversity became lower, and *Haemophilus*, *Streptococcus*, and *Neisseria* were the dominant microflora which had higher abundance compared with the healthy state. S.pn, HI, M.ca and SA were dominant profiles in the included studies, but the initially homogeneous microflora rapidly diversified across body sites under local pressures and age. For example, the nasopharyngeal microflora develops rapidly in the first weeks of life, with SA emerging as the core flora, then followed by colonization and outgrowth of *Corynebacterium* and *Dolosigranulum*. M.ca rapidly started to populate and ultimately overtake this niche by the age of 3 months [4 8 10 11 17]. A variety of bacterial flora are colonized rapidly in the respiratory tract in infants postnatally and their abundance changes with age. In particular, it has been shown that Moraxella catarrhalis peaks at 3 months of age.

### 3.4 The effect of different dominated microflorae with RSV infectious on disease severity

The short‐term outcomes of RSV infectious disease with dominated microflorae in different studies are presented in Table 1.

**TABLE 1:** Main findings of the original articles indicating the microflora colonization on RSV disease severity (*n* = 16).

Study	Region	Date	Disease	Dominant profiles	Sample source	Short‐term outcome
Embriette R Hyde et al^10^	America	2014	Bronchiolitis	HI, M.ca	NPA	Compared with solo RSV infection, RSV and HI‐M.ca co‐infection had a significantly longer length‐of‐stay.
Kohei Hasegawa et al^6^	America	2016	Severe bronchiolitis	S.pn, M.ca, HI, *Prevotella* and SA	NPA	1) HI dominated profile had the highest intensive care use rate and M.ca dominant profile was the lowest. HI dominant profile had a higher rate of length of stay of ⩾3 days than M.ca dominant profile. 2) HI dominated profile had a negative effect on RSV disease severity.
Wouter A.A.de Steenhuijsen Piters et al^11^	The Netherlands	2016	RSV disease	HI, S.pn, SA, M.ca, *Corynebacterium*	NPA	Infants with RSV diseases had a higher abundance of HI and S.pn dominant profile. Data suggests that the disease severity between clusters weren't significant.
Marloes Vissers et al^16^	The Netherlands	2016	RSV infection	S.pn	NPA	Presence of S.pn does not correlate with disease severity. It's the first to show a negative correlation between RSV disease severity and S.pn density and suggest that high S.pn density protects against severe infections. This suggests that severely ill infants have lower RSV levels, which seems contradictory.
Kohei Hasegaw et al^12^	America	2017	Severe bronchiolitis	HI, M.ca, S.pn	NPA	1) HI‐dominated profile was associated with a higher risk of intensive care use compared to *Moraxella* dominant profile. 2) The specific function of airway microbiota influences bronchiolitis severity by altering the expression of CCL5(C‐C Motif chemokine Ligand 5) locally.
Kohei Hasegaw et al^13^	America	2017	Bronchiolitis	HI, M.ca, S.pn	NPA	Among infants with lower LL‐37 (the main active form of human cathelicidin) levels, HI dominant profile was associated with higher risks of intensive care use compared to *Moraxella* dominant profile.
Christopher J. Stewart et al^3^	America	2017	Bronchiolitis	HI, M.ca, S.pn,	NPA	Abundance of S.pn was positively correlated with majority of metabolites predicting high risks of PPV (positive pressure Ventilation) use. S.pn was positively related with RSV disease severity.
Jaelle C. Brealey et al^20^	Australia	2017	RSV ARI	S.pn	NPA	RSV and S.pn codetection were associated with high RSV disease severity.
Christian Rosas‐Salazar et al^2^	America	2018	RSV ARI	M.ca, S.pn, HI, *Corynebacterium*, *Dolosigranulum*.	NPA	The relative abundance of *Lactobacillus* was negatively associated with respiratory severity generally among infants with subsequent wheeze.
Thomas H. A. Ederveen et al^21^	The Netherlands	2018	RSV infection	HI, S.pn, M.ca, *Corynebacterium*, SA, *Prevotella*, *Achromobacter*, *Neisseria*, *Veillonella*	NPA	HI abundance is the strongest predictor for CXCL8^a^ levels. The nasopharyngeal flora in young infants with RSV infection is marked by an overrepresentation of the genus HI.
Jonathan M. Mansbach et al^22^	America	2019	Severe RSV bronchiolitis (need hospitalization)	HI, M.ca, S.pn and mixed profiles	NPA	The primary outcome: Delayed clearance.^b^ RSV bronchiolitis with nasopharyngeal microbiota is related to delayed RSV clearance which suggests that it may be positively correlated with RSV disease severity.
Abhijeet R. Sonawane et al^23^	America	2019	RSV infection	M.ca, *Corynebacterium*, S.pn, HI, SA and *Ralstonia*.	Nasal sample	HI was positively associated with severity, but *Ralstonia* and S.pn as negatively associated with disease severity.
Christopher J. Stewart et al^24^	America	2019	Bronchiolitis	S.pn	NPA	1) The primary outcome: The use of PPV. Relative abundance of S.pn was positively correlated with metabolites associated with a higher risk of PPV use. Abundance of M.ca was negatively with PPV use. 2) RSV infection with S,pn detection was positively associated with disease severity.
Christian Rosas‐Salazar et al^15^	America	2021	RSV infection	*Klebsiella*, HI, S.pn, *Methylobacterium*	Nasal sample	Respiratory ⍺‐diversity^c^ was positively associated with an increased RSS, greater odds of a lower ARI.
Alejandro Diaz‐Diaz et al^25^	America	2022	RSV infection	S.pn, HI, M.ca, SA	NPA	1) Oxygen administration was more frequent in the codetection of S.pn and HI. 2) Children with RSV infection had higher CDSS with any bacteria colonization, except SA. Children with SA detection had the lowest CDSS compared to children with other bacteria. Children with RSV infection with S.pn, HI codetection had higher CDSS compared with no detection. 3) Children with RSV infection with S.pn, HI codetection increased rates of PICU admission. 4) Children with RSV infection with S.pn, HI was positively associated with RSV disease severity.
Long Xin et al^19^	China	2022	Pneumonia	S.pn	NPA	The presence of S.pn dominant profile may aggravate the severity of RSV infection in children age ≥6m.

Abbreviation: S.pn, HI, M.ca, SA, Streptococcus pneumoniae, Haemophilus influenzae, Moraxella catarrhalis, Staphylococcus aureus.

^a^ CXCL8 (C‐X‐C Motif Chemokine Ligand 8): Nasopharyngeal microflorae are significantly different based on CXCL8 levels. CXCL8 is a chemokine that was previously found to be indicative for disease severity.

^b^ Delayed clearance: An infant having the same RSV subtype at hospitalization and 3 weeks after the date of hospitalization.

^c^ α‐diversity: Within‐sample measures of the richness (the total number of different microbial species) and evenness (relative differences in the abundance of various species) of a community of microbes in an ecological niche (e.g., genus richness, Shannon diversity index and Simpson index).

From the summary of effect of bacterial colonization on RSV disease severity, RSV infectious disease severity was assessed by a CDSS or RSS, the need of supplemental oxygen, PICU admission and length‐of‐stay. HI, M.ca, S.pn, SA are the dominant profiles in the respiratory tract with RSV infection in children from NPA. Different studies had inconsistent outcomes which mainly concentrated on the following:

For S.pn dominant profile: Presence of S.pn doesn't correlate with disease severity, but S.pn density was associated with titers of RSV. The children who had more severe RSV disease had lower S.pn densities. It is the first study to illustrate that S.pn had a negative effect on RSV disease severity^16^ and Abhijeet R.Sonawane et al^23^ also found that S.pn was negatively associated with disease severity. The data showed that severely ill infants have lower RSV levels, which seems contradictory. A possible explanation for this discrepancy is that at the moment the infants are included, they are in an advanced stage of disease. It is therefore possible that in the severe cases the virus has already partly been cleared and that severe inflammation is the cause of the severity, not viral load. Another explanation is that the interactions between S.pn, RSV load, inflammation, and disease severity are multidirectional and more complex than we can grasp in the study. Otherwise, Christopher J. Stewart and other researchers^3^, ^19^, ^20^, ^24^, ^25^ showed that S.pn abundance was positively correlated with disease severity.
For HI dominant and M.ca dominant profiles: Compared with solo RSV infection, RSV and HI, M.ca co‐detection had a significantly longer length‐of‐stay.^10^ Compared with *Moraxella*‐dominant profile, HI dominant profile had a longer length‐of‐stay and intensive care unit especially with Lower CCL5 or LL‐37 in serum.^6^, ^12^, ^13^ Christopher J. Stewart et al^24^ suggested that HI was positively associated with disease severity.
For SA dominant profile: Alejandro Diaz‐Diaz et al. found that Children with RSV infection had higher CDSS with any bacteria colonization, except SA.^25^

### 3.5 The effect of dominated microflorae with RSV infectious on recurrent wheezing or childhood asthma

The long‐term outcomes of RSV infectious disease with dominated microflorae in different studies are presented in Table 2.

**TABLE 2:** Main findings of the original articles indicating the microflora colonization on childhood wheezing (*n* = 5).

Study		Region	Date	Disease	Dominant profiles	Sample source	Long‐term outcome
Christian Rosas‐Salazar et al^2^		America	2018	RSV ARI	M.ca, S.pn, HI, *Corynebacterium*, *Dolosigranulum*, *Pseudomonas*.	Nasal sample	Outcome: 2‐year subsequent wheeze and recurrent wheeze.^a^ 1) Increased abundance of *Lactobacillus* was associated with a reduced risk childhood wheezing illnesses at age 2 years. 2) Data showed detection of *Lactobacillus* in RSV‐infected infants could be used as a biomarker for the later development of childhood wheezing illnesses.
Jonathan M. Mansbac et al^22^		America	2019	Bronchiolitis	M.ca, HI, S.pn	Nasal sample	Outcome: Recurrent wheezing by age 3 years. 1) 88 percent of 842 infants hospitalized for bronchiolitis followed up at 3 years and 31% developed recurrent wheezing. 2) Abundance of M.ca or S.pn was associated with an increased risk of recurrent wheezing.
Yoshihiko Raita et al^14^		America	2019	RSV bronchiolitis	*M. Nonliquefaciens*, S.pn, M.ca	NPA	The primary outcome: Asthma at age 5 years. The secondary outcome: Development of recurrent wheeze by age 3 years. 1) Compared with M. nonliquefaciens dominant profile, S.pn and M.ca dominant profiles had a significantly higher risk of developing asthma by age 5 years and recurrent wheeze by age 3 years.
Xiaoyan Zhang et al^26^		China	2020	Severe RSV bronchiolitis	S.pn HI, M.ca, unidentified *Prevotellaceae*, and unidentified *Clostridiales*	NPA	1) Abundance of *Haemophilus*, *Moraxella*, and *Klebsiella* was higher in infants who later developed recurrent wheezing than in those who did not. 2) Data suggested that higher abundance of *Haemophilus* and *Moraxella* in airway microbiome might modulate airway inflammation during severe RSV bronchiolitis in infancy, potentially contributing to the development of subsequent recurrent wheezing in later childhood.
Christian Rosas‐Salazar et al^15^	America		2021	RSV infection	*Klebsiella*, HI, S.pn, *Methylobacterium*	Nasal sample	Richness of the upper respiratory tract microflora was not associated with the number of wheezing episodes in the fourth year, but ⍺‐diversity was positively associated with higher number of wheezing episodes in the fourth year of life.

^a^ Recurrent wheeze: Defined as parental report of ≥2 episodes of wheeze since the last birthday.

### 3.6 The effect of different dominated microbiomes on childhood asthma or recurrent wheezing with RSV infection

From the five articles which mentioned the long‐term outcomes, it seemed that *Lactobacillus* and *Staphylococcus* were a protective factor in subsequent wheeze.^2^ On the contrary, abundance of M.ca, HI and S.pn was associated with an increased risk of recurrent wheezing.^14^, ^26^, ^27^ It needs more studies to further research the association between respiratory microflorae and RSV infection, and make contributions to the field of it.

## 4 DISCUSSION

The concept of respiratory microflorae colonization was recognized in the decade and there existed significant room for improvement to expand the diversity of types and designs of studies. Most included studies were carried out in developed countries and it should be treated as a call for action for developing countries. RSV is a global health burden which induces major mortality and susceptibility to severe RSV bronchiolitis is governed by gene‐environmental‐host and microflora interactions which affect the host response to RSV infection. Interactions between respiratory microflorae and host immune response may present a tipping point in the balance of disease severity and long‐term outcomes.^28^ Researchers postulated that perturbed microflorae composition results not only in diminished colonization resistance and symptomatology during RTIs, but also in persistence of risk recurrences. The exact mechanisms have not been illustrated. Specific microbial communities may promote pro‐inflammatory milieu and facilitate blooms of pathogens.^29^

The result of our review indicated that respiratory microflorae colonization during RSV infection was associated with disease severity and asthma or recurrent wheezing in childhood, but results were inconsistent across studies. Viral clearance, vaccination rate, daycare attendance and presence of siblings could influence pneumococcal carriage patterns. Theoretically, more severe RSV infected infants had a higher pneumococcal density instead of a lower load. However, severe RSV infected infants had no chance to receive pneumococcal vaccination. Brand, HK et al^30^ showed that patients with severe infection had more RSV mono‐infections compared to other severity groups, so it made researchers not exclude whether less daycare attendance or other factors in severely infected children may have contributed to lower pneumococcal carriage. On the contrary, S.pn colonization enhanced RSV replication in human bronchial epithelial cells and preceding RSV infection predisposed to invasive pneumococcal disease in animal model.^31^ Epidemiologic studies showed a relationship between peak activity of RSV and incidence of invasive pneumococcal disease and reduction in pneumococcal carriage by vaccination led to reduction of RSV hospitalization.^32^, ^33^

Thomas Gensollen et al^34^ discussed that the immune influences induced by microflorae during early life may be durable, creating a “window of opportunity” for immune education to occur and resistance to disease in later life, but a significant caveat remains the sparsity of proven casual links. Madeleine F. Jennewein et al^35^ showed that the time discrepancy between the first deviation microflorae development and the occurrence of respiratory tract infections (RTIs) which occurs typically from 4 months on, indicating susceptibility to RTIs could be at least partially due to other outcomes with timing of microflorae colonization.^35^ Critical for the association between microflorae development and RTI susceptibility appears to be timing of colonization events. Evidence have shown that in the first 3 months of life, *Staphylococcus* aureus‐dominated profile rapidly colonized and replaced by a *Moraxella*‐dominated microbiota at age of 3–6 months whose early emergency was related to an immune response of airway mucosa and later development of asthma or recurrent wheezing.^36^ In addition, enrichment of S.pn before 9 weeks was related to a higher risk of early RTI.^37^ Nowadays, there exists an increasing interest in understanding the interplay between host and microflorae. Data has corroborated the association of M.ca and S.pn abundance to childhood wheezing outcomes.^38^ For infants hospitalized with bronchiolitis, the “window of opportunity” to influence the secondary succession of the nasal microbial ecosystem, may potentially be the risk of developing recurrent wheezing by the age of 3 years or asthma at the age of 4 years.^39^, ^40^

Apart from interaction between host, respiratory microflorae and RSV infection, studies have demonstrated that modulating gut microflorae may potentially reduce the morbidity of viral acute respiratory infections.^41^, ^42^ Leyer, G.J et al^43^ reported that the use of *Lactobacillus* acidophilus reduced acute respiratory infections symptoms. Jeffrey N. Harding et al^44^ showed that infants with severe RSV infection had slightly lower *α*‐diversity of the gut microbiota compared to patients with moderate RSV and healthy controls. Overall, these studies demonstrated that gut microflorae was associated with RSV disease and with admission to the pediatric intensive care unit in severe RSV infected children.^45^, ^46^, ^47^, ^48^, ^49^ An animal study showed that gut microflorae colonization especially in the neonatal window may protect from ovalbumin‐induced accumulation of invariant natural killer T (iNKT) cells into the lungs, and it's a process related to the development of allergic asthma.^50^ A birth cohort demonstrated that there existed a time link between early abundance of *Bifidobacterium spp*. and the number of RTIs during the children born by vaginal birth. The study suggested that gut microflorae may serve as a mediator between known risk factors (such as mode of delivery) and subsequent RTI susceptibility.^51^

We also found that metatranscriptomics was commonly used to capture the respiratory virome, microflorae, and host responsed directly from low biomass samples. To date, there were only a few studies focused on researching the entire human respiratory virome during health or disease.^52^ Seesandra V. Rajagopala et al^53^ revealed that HI was highly active in symptomatic RSV subjects. Current pilot study provides support for the use of a transcriptomic approach to delineate the integrated contributions of the airway microbiome and host to bronchiolitis pathobiology.^14^, ^54^, ^55^

In summary, “one pathogen‐one disease” pattern has started to shift toward an overall ecosystem of RTI pathogenesis. In order to figure out the relationship between host, microflorae, gene and environment, more and more studies created the integrated microbiome and host transcriptome analysis.^56^ Revealing the association between microbiota composition, function and the pathogenesis of RSV disease is crucial, but an important caveat remains the sparsity of proven causal links. The major challenge of the microbiome field is to get the complexity of ecosystem‐wide host‐microbe interactions which could represent the human organism in model systems.^57^

In this study, (1) we noted the prevalence of the microflorae with RSV infection differs in regions. This imbalance could be a result of different economies, medical conditions, policies, and recognition of the concept. (2) It is essential to expand the diversity of types and design of studies. (3) We summarized the effect of dominated profiles in the respiratory tract on disease severity and childhood asthma. This study has certain limitations. (1) A certain proportion of conference abstracts informing the data were included. (2) The articles included were small, and the results were not necessarily accurate due to the difficulty of harmonizing data across articles.

## 5 CONCLUSION

Over the last couple of years, the general “a pathogen‐a disease” pattern has started to shift toward an ecosystem‐wide theory of RTI pathogenesis and its theory included the interactions between gene‐host‐environment and microflora mediated immune modulation and susceptibility. This scoping review presents the state of the studies published or posted online which were related to the effect of dominant profiles in respiratory tract on RSV infectious disease severity and childhood asthma or recurrent wheezing. The studies in this field are increasing in the decade, but they are mainly concentrated in developed countries. Microbial exposure in the respiratory tract, such as HI, M.ca, S.pn may contribute severe to RSV infectious disease compared with mild RSV disease, but the outcome in this scoping review is not consistent. On the other hand, the evidence is also insufficient to suggest accurate targeted interventions, although some directions have been mentioned, such as the microbiome influences the disease severity, the development of the respiratory tract in children, and the long‐term prognosis of wheezing disease, but more studies should be designed to figure out the accurate correlation.

## AUTHOR CONTRIBUTIONS

Lidan Gan analyzed and interpreted the data, and critically revised the manuscript for important intellectual content; Yu Deng conceived and designed the work, drafted the initial manuscript, and revised the manuscript; Prof Enmei Liu carried out the initial analyses and revised the manuscript. All authors have read and agreed to the published version of the manuscript.

## CONFLICT OF INTEREST STATEMENT

The authors declare that there is no conflict of interest.

## ETHICS STATEMENT

Not applicable.

## PATIENT CONSENT STATEMENT

Not applicable.

## Supporting information

Supporting Information S1

## Data Availability

The data that supports the findings of this study are available in the supplementary material of this article.
